# Estimated dengue force of infection and burden of primary infections among Indian children

**DOI:** 10.1186/s12889-019-7432-7

**Published:** 2019-08-14

**Authors:** Amit Bhavsar, Clarence C. Tam, Suneela Garg, Guru Rajesh Jammy, Anne-Frieda Taurel, Sher-Ney Chong, Joshua Nealon

**Affiliations:** 1 Sanofi Pasteur- India, Mumbai, India; 2 Present address: Saw Swee Hock School of Public Health, National University of Singapore and National University Health System, Singapore, Singapore; 3 London School of Hygiene & Tropical Medicine, London, UK; 4 Maulana Azad Medical College, Bahadur Shah Zafar Marg, New Delhi, Delhi, 10002 India; 5 SHARE INDIA – Mediciti Institute of Medical Sciences, Hyderabad, India; 6 Sanofi Pasteur- Singapore, Asia & JPAC, 38 Beach Road # 18-11, South Beach Tower, Singapore, 189767 Singapore; 7 Present address: GSK Biologicals, Rixensart, Belgium

**Keywords:** Dengue, Endemic diseases, Flavivirus, India, Infection, Seroepidemiologic studies

## Abstract

**Background:**

Comprehensive, age-stratified dengue surveillance data are unavailable from India and many more dengue cases occur than are reported. Additional information on dengue transmission dynamics can inform understanding of disease endemicity and infection risk.

**Methods:**

Using age-stratified dengue IgG seroprevalence data from 2556 Indian children aged 5–10 years, we estimated annual force of infection (FOI) at each of 6 sites using a binomial regression model. We estimated the ages by which 50 and 70% of children were first infected; and predicted seroprevalence in children aged 1–10 years assuming constant force-of-infection. Applying these infection rates to national census data, we then calculated the number of primary dengue infections occurring, annually, in Indian children.

**Results:**

Annual force-of-infection at all sites combined was 11.9% (95% CI 8.8–16.2), varying across sites from 3.5% (95% CI 2.8–4.4) to 21.2% (95% CI 18.4–24.5). Overall, 50 and 70% of children were infected by 5.8 (95% CI 4.3–7.9) and 10.1 (95% CI 7.4–13.7) years respectively. In all sites except Kalyani, > 70% of children had been infected before their 11th birthday, and goodness-of-fit statistics indicated a relatively constant force-of-infection over time except at two sites (Wardha and Hyderabad). Nationwide, we estimated 17,013,527 children (95% CI: 14,518,438- 19,218,733), equivalent to 6.5% of children aged < 11 years, experience their first infection annually.

**Conclusions:**

Dengue force-of-infection in India is comparable to other highly endemic countries. Significant variation across sites exists, likely reflecting local epidemiological variation. The number of annual primary infections is indicative of a significant, under-reported burden of secondary infections and symptomatic episodes.

**Trial registration:**

Registered retrospectively with clinicaltrials.gov (NCT01477671; 18/11/2011) and clinical trials registry of India (ctri.nic.in; CTRI/2011/12/002243; 15/12/2011). Date of enrollment of 1st subject: 22/9/2011.

**Electronic supplementary material:**

The online version of this article (10.1186/s12889-019-7432-7) contains supplementary material, which is available to authorized users.

## Background

Dengue has become hyperendemic in many parts of India [1, 2]. The disease is being reported from an increasing number of states, and the number of cases reported to the National Vector Borne Disease Control Program (NVBDCP) has been increasing over recent years. In 2010, the incidence of reported dengue was 2.3 cases per 100,000 individuals, increasing to 11.7 per 100,000 in 2017 [3]. In 2016, for the first time, more than 100,000 cases were reported (total: 129,166 with 245 deaths). However, reported cases represent only the tip of the iceberg, and the true disease burden is likely significantly higher [4]. Mild cases are particularly susceptible to under-reporting [5]. Notably, a global cartographic modeling study by Bhatt et al. provided comprehensive global dengue burden estimates, and projected > 32 million cases in India in 2010 [6]. A complementary study by Stanaway and colleagues from the Institute for Health Metrics and Evaluation, using verbal autopsy, vital registration and surveillance data estimated 18.6 million cases in 2013 [7]. A local estimate focusing on the city of Chennai (population of 4.7 million) used seroprevalence data to estimate 89,700 new infections and 138,100 secondary infections every year [8]. This distinction is important because dengue has four serotypes; and second infections are more commonly severe [2].

In the absence of incidence data including cases which were not recognized as dengue and those who did not access healthcare, seroprevalence data provide an alternative indicator of transmission intensity. [9] Seroprevalence describes historical infection and, when derived with standardized diagnostics, is a relatively unbiased indicator of viral exposure when compared with surveillance data. Age-stratified surveys provide data from which one can derive force of infection (FOI) estimates and therefore understand the infection rate [10, 11]. Understanding endemicity is important for a wide range of public health decision-making and, given that the world’s first dengue vaccine’s efficacy is associated with baseline serostatus, population level seroprevalence is an important predictor of population-level vaccine impact [12].

In India, as elsewhere, few studies have documented the seroprevalence of dengue in healthy subjects. In the earliest, Padbidri et al. measured exposure to various arboviruses, including dengue serotype-2, in the Andaman and Nicobar Islands. This 1988–89 study found 25.4% of subjects with neutralizing antibodies against dengue type-2 [13]. More recently, Oruganti et al. examined the presence of antibodies in healthy individuals attending routine health check-ups in Hyderabad, Andhra Pradesh by indirect IgG ELISA [14]. They found 89.5% of subjects aged 19 to 70 years of age were seropositive for dengue: 100% of those 40 years of age or older had seroconverted. In another community-based study Rodriguez et al. estimated seroprevalence in 5–40 year old healthy subjects in 2011 in Chennai [8] They demonstrated that 93% of subjects in this age group had been exposed to dengue at least once in their lifetime, a level of exposure which was consistent with long-term endemic circulation.

We previously published results of a community-based multi-centric, cross-sectional study (DNG10) on dengue seroprevalence in Indian children aged 5–10 years (CTRI/2011/12/002243 and NCT01477671) [15]. The study was conducted at 8 sites in 6 distinct urban and rural areas in 2011–12. Overall seroprevalence was 59.6% and increased with age. We also described monotypic serological profiles demonstrating that all four dengue serotypes circulate in India.

No previous analysis has assessed dengue FOI and its variability across multiple Indian sites. Here, we conducted a secondary analysis to estimate dengue FOI in healthy children in different geographic regions of India. In combination with census data, this enabled estimation of the number of primary dengue infections occurring annually. We also predicted seroprevalence in children aged 1–10 years of age and the ages at which 50 and 70% of children have experienced at least 1 dengue infection, to inform vaccination policy.

## Methods

### Ethics statement

As this was secondary analysis, no additional ethical approvals were needed. Details of ethical approvals for the original study are provided in Garg et al. [15].

### Source of dengue seroprevalence data

DNG10 was a dengue seroprevalence study which collected blood samples from children aged 5–10 years old between January 2011 and October 2012. There were 8 sites across 6 districts spread over India (two nearby sites each from Delhi and Hyderabad; and one site each from Kalyani, Wardha, Mumbai and Bangalore), which have been described before [15]. Briefly, a convenience sample of children was drawn from the community by household visits (6 sites) or school visits (2 sites). Community health workers obtained informed consent and drew blood samples. The presence of anti-dengue IgG antibodies was measured using one of two commercial ELISA (Focus Diagnostics, California, USA and Panbio Diagnostics, Brisbane, Australia) whose performances were shown to be concordant [15]. We performed a reanalysis of data from this original study after pooling data from the two Delhi and Hyderabad sites, assuming that populations in these sites were exposed to a similar risk of infection because of their geographical proximity (within a few hundred meters).

### Force of infection and seroprevalence estimates

**Fig. 1:**
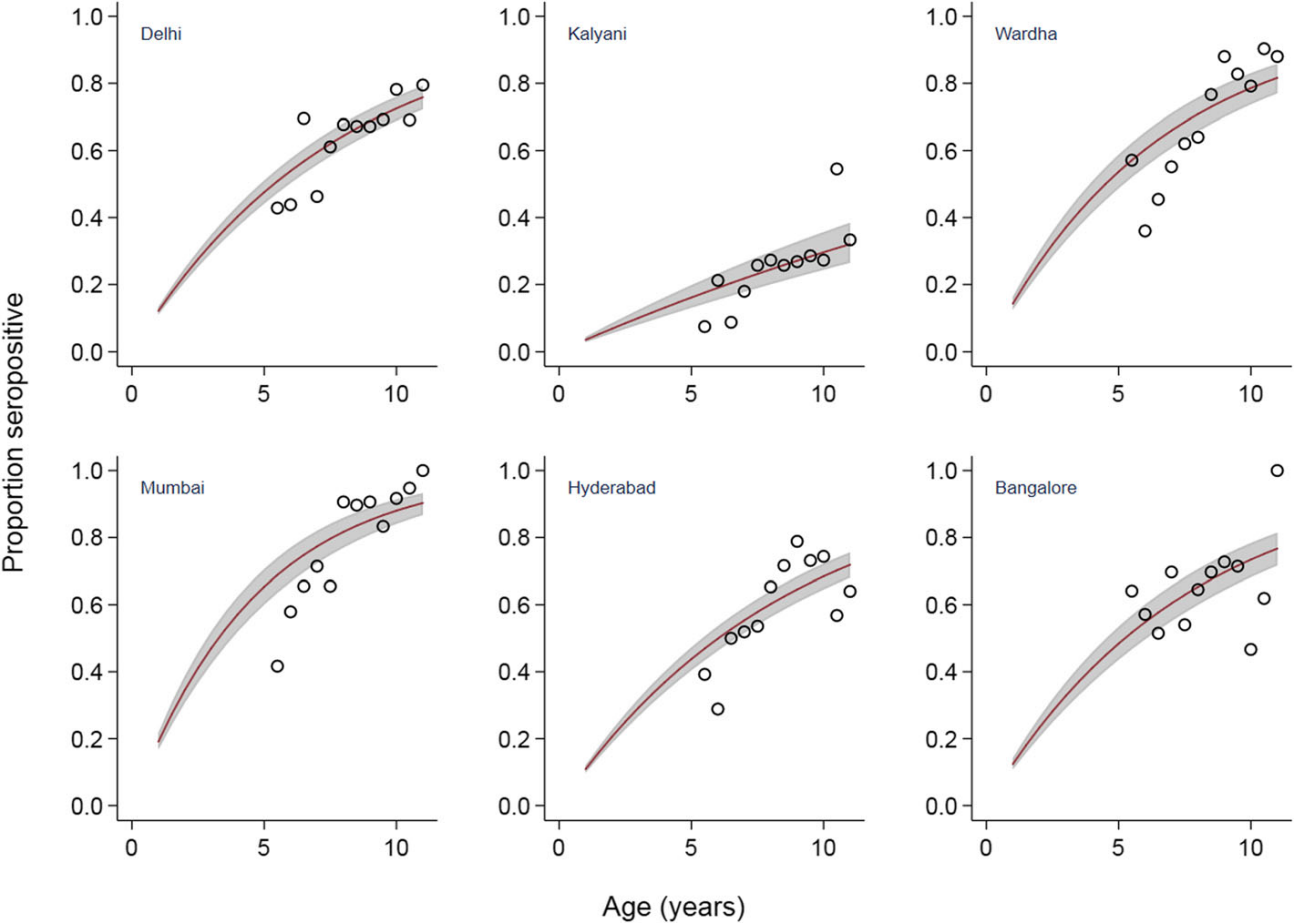
Estimated seroprevalence (red lines), 95% confidence intervals (shaded areas) and observed seroprevalence (circles)*. *hollow circles indicate observed seroprevalence as measured in original study, divided into 6-month age categories [15]

Dengue serostatus was considered a binary outcome variable, described by the IgG ELISA test result for each subject and assuming seroconversion is non-reversible. Assuming constant FOI over this 6-year age group, we estimated FOI (λ) using a catalytic model which predicts an increase in the proportion of seropositive individuals with age: [10, 16].

$$ {p}_a=1-{e}^{-\lambda a} $$

where *p*_*a*_ is the proportion seropositive at age *a*. We estimated λ using a binomial regression model with a complementary log-log link, including seropositivity as the outcome variable and the natural logarithm of age as an offset, a parametrization in which the constant equals the log of average FOI [16, 17]. Separate estimates were made for each site; and for all sites combined. Clustering both at the national level, and for Delhi and Hyderabad where two sites were combined, was accounted for by relaxing the assumption of independence of observations within groups and generating robust standard errors. Seroprevalence and its 95% confidence intervals for children aged from 1 to 10 years old were estimated from FOI using the formula above. We estimated the ages “*a*” at which prevalence “*P*” was 0.5 and 0.7, and their confidence intervals, using the same formula and by replacing “λ” with the estimated constant FOI from each site. The six years of age groups of observed seroprevalence data were grouped into 12, 0.5 year age categories. Mean seroprevalence for each group was graphed over the estimated seroprevalence, as shown in Fig. 1.

### Force of infection and seroprevalence estimates

Based on Indian 2011 census data [18] and estimated annual seroconversion rates, we estimated the number of children aged < 11 years experiencing a primary dengue infection in 2011, assuming constant FOI from 2002 to 2011, according to the formula below:

$$ \sum \limits_{a=1}^{11}{\delta}_a\left({p}_a-{p}_{a-1}\right) $$

Where, *δ*_*a*_ represents the total size of the Indian population aged *a* years; and *p*_*a*_ is the proportion of the population seropositive by age *a* years.

### Assessment of model fit

In our model, we assumed a constant force of infection. Goodness of fit was assessed by the Hosmer-Lemeshow test [19]. The predicted probabilities of being seropositive and seronegative were calculated for each individual and the data were grouped into deciles. The expected number of events, calculated as the sum of the predicted probabilities, was compared with observed events. Pearson’s chi-squared test was applied to test the null hypothesis that the observed data approximates the fitted model under an assumption of constant FOI, with a *p*-value of > 0.05 applied to define an acceptable fit.

All analyses were conducted with Stata version 15.0 (Stata Corporation) and Microsoft Excel.

## Results

### Demographics of study subjects and observed seroprevalence

In total, the analysis included data from 2556 subjects, with between 301 and 649 children per site, with approximate equal age distributions (Table 1, see Additional file 1 for detailed age distributions). 52.6% of the subjects were female and the mean age of participants was 7.8 years (SD 1.6 years) with a range 5.0–10.0 years.

**Table 1:** Number of subjects, mean age and overall seroprevalence by site [15]

Site name	Number of subjects enrolled	Mean age in years (standard deviation)	Seroprevalence (%) (95% CI)
Delhi (for 2 sites)	649	8.0 (1.7)	63.3 (59.6–67.0)
Kalyani	323	7.6 (1.4)	23.2 (18.7–28.2)
Wardha	323	8.0 (1.7)	69.0 (63.7–74.0)
Mumbai	301	8.0 (1.5)	80.1 (75.1–84.4)
Hyderabad (for 2 sites)	639	7.8 (1.6)	58.4 (54.5–62.2)
Bangalore	321	7.5 (1.5)	62.6 (57.0–67.8)
Total	2556	7.8 (1.6)	59.6 (57.7–61.5)

### Estimated force of dengue infection

The overall annual FOI for all sites combined was 11.9% (95% CI 8.8–16.2%). It varied from a low of 3.5% (95% CI 2.8–4.4%) in Kalyani, West Bengal, to 21.2% (95% CI 18.4–24.5%) in Mumbai, Maharashtra (Fig. 1). Assuming constant FOI, the ages by which 50 and 70% of children were first infected were lowest in Mumbai, 3.3 and 5.7 years respectively (Table 2). In Kalyani FOI was sufficiently low that we predicted < 50% of children would have been infected by the age of 11. For other sites, the median age of infection was between 3.3 and 6.0 years; 70% of children were estimated to have been infected by between 5.7 and 10.4 years of age. In the study population overall, 70% of children were estimated to have been infected at least once by the age of 10.1 years. Model goodness of fit as assessed by the Hosmer-Lemeshow test was acceptable for all sites except Wardha (*P*-value: 0.03), Hyderabad (*P* = 0.01) and for India overall (P = 0.01).

**Table 2:** Annual FOI, goodness-of-fit statistics; and the ages by which 50 and 70% of children seroconverted

Site	Annual FOI, % (95% CI)	Goodness of fit Chi^2^ statistic; P- value	Age of 50% population seroconversion, years (95% CI)	Age of 70% population seroconversion, years (95% CI)
Delhi (2 sites)	12.9 (11.1–15.0)	2.96; 0.94	5.4 (4.6–6.2)	9.3 (8.0–10.8)
Kalyani	3.5 (2.8–4.4)	4.72; 0.79	> 11	> 11
Wardha	15.4 (13.4–17.7)	16.9; 0.03	4.5 (3.9–5.2)	7.8 (6.8–9.0)
Mumbai	21.2 (18.4–24.5)	12.8; 0.12	3.3 (2.8–3.8)	5.7 (4.9–6.6)
Hyderabad (2 sites)	11.5 (11.2–11.8)	20.9; 0.01	6.0 (5.9–6.2)	10.4 (10.2–10.7)
Bangalore	13.2 (11.5–15.3)	10.2; 0.25	5.2 (4.5–6.0)	9.1 (7.9–10.5)
All sites combined	11.9 (8.8–16.2)	20.4; 0.01	5.8 (4.3–7.9)	10.1 (7.4–13.7)

### Estimated number of primary dengue infections

In 2011, India had a population of ~ 260,000,000 children aged < 11 years. We estimate that in 2011 17,013,527 (95% CI 14,518,438 – 19,218,733) children aged up to 10 years – 6.54% of the total population within this age group – were infected with dengue for the first time (see Additional file 2).

## Discussion

We conducted a secondary analysis of dengue seroprevalence data from pediatric populations in India. We found that among dengue-naïve children, 11.9% experience their first dengue infection every year. This means that 50% of children at these sites are infected by dengue at least once by the age of 5.8 years, and 70% of them are infected by the age of 10.1 years, although there was significant variation in FOI between sites. Our study was not the first to report estimates of dengue FOI in Indian populations. Imai et al. used data from 1988 to 89 to estimate FOI of 0.2% (95% CI: 0.1–0.7%) in the Andaman and Nicobar Islands [11, 13]. Rodriguez et al. estimated that the dengue FOI in Chennai from 2004 to 2011 was 23% (95% CI: 16–30%) [8]. The Andaman and Nicobar Islands are a unique geography; that study detected antibodies against only one of the four serotypes of dengue (dengue serotype- 2), and was conducted at a time when dengue endemicity was probably much lower than today. Rodriguez et al. sampled probabilistically from Chennai and found high FOI in pediatric populations. We identified similar FOI from Mumbai, a city with similar ecological conditions: both are coastal with similar ranges of temperature and high levels of unplanned infrastructure, construction sites and slum housing.

We assumed these sites experienced constant FOI for the 5 years prior to sample collection, representing the time period when study subjects were infected. A different approach would consider FOI to be time-varying, in which constant FOI is assumed only for a certain period [9]. Our assumption is broadly consistent with other studies that have found age-constant models adequately describe age-related seroprevalence data over a 6–9 year time horizon [8, 10]. The goodness-of-fit of our constant model provided some evidence that our assumption of constant FOI is valid for four of our six sites, but to more completely explore age-varying FOI, data from a larger age range of subjects would be needed. Further, a visual inspection of Fig. 1 suggests some deviation between the modelled values and the observed data especially at more extreme ages. This may be due to cyclical dengue outbreaks in the respective geographies. For example, there were documented outbreaks in Mumbai in 2003, and in Wardha and Hyderabad in 2004 [20–22]. Children were disproportionately affected in Mumbai and Wardha which might provide an explanation for outliers in our observations i.e. higher seroprevalence in older children at these sites.

Similar dengue FOI has been estimated from seroprevalence data from dengue hyperendemic Southeast Asian countries. Prayitno et al. estimated the FOI in 1–18 year old Indonesian children in 2014 to be 14.0% [23]. Imai et al. estimated FOI in Thailand using data from 2000 to 01 in school children to be 15.7% [11]. Using 2008–09 data in children under 12 from Colombo, Sri Lanka, Tam et al. estimated the FOI to be 14.1% [10]. Consequently, and because reported dengue incidence rates in India are so low, we calculated the resulting number of primary dengue infections, estimating > 17 million primary infections in India, annually. Other researchers have estimated between 30 and 50% of primary infections are symptomatic [24] which would equate to ~ 5 – ~ 8.5 million cases annually in children aged < 11. When considering cases in other age groups, and following secondary or subsequent infections; these case numbers are broadly within the same range as those reported in Bhatt et al., that India suffers ~ 35 million symptomatic episodes per year, and provide additional evidence of a very significant level of under-reporting of dengue in India [6]. More detailed estimates of symptomatic episodes are limited by our lack of secondary infection history data; and more complex mathematical modeling was beyond the scope of our study.

This is the first study to estimate FOI in India using data from multiple geographies; urban and rural, and from multiple states. Our results point towards a high dengue FOI in children in India, which logically equates to a significant number of secondary infections and burden of symptomatic disease in this age group. With improved surveillance, we may begin to see incidence rates of dengue in India comparable to those seen in other hyperendemic countries. Longitudinal cohort studies, ideally incorporating fever surveillance and serological surveys, to more accurately describe the incidence of dengue and changing infection patterns with age, are needed [25].

Our study has several limitations. The original seroprevalence samples were collected in 2011–12 and the FOI we have derived corresponds to cumulative exposure experienced by study subjects in the years of their life before this time. Numbers of reported cases of dengue in India have increased significantly from 2011. [3] This can be attributed to several factors including population movement and increased exposure to the virus; improved dengue surveillance, increasing awareness among healthcare practitioners, availability of confirmatory diagnostics and improvement in access to healthcare resulting in increased reporting [4, 26]. As demonstrated by Rodriguez et al.in Chennai, it is also very likely that FOI has increased in India over recent decades [8]. Despite their geographical spread, study sites were not sampled to be representative of the whole of India and our extrapolation to the national level is a strong assumption which should be validated with more recent data from other sites. DNG10 also used convenience sampling for enrollment of subjects, a method which does not guarantee representativeness. We used IgG ELISA to ascertain infection history, an assay with known cross-reactivity to antibodies against other flaviviruses. However, dengue infection was confirmed by the plaque reduction neutralization test (PRNT) and > 97% of IgG positive samples were also positive by PRNT. Further, Japanese encephalitis (JE) seropositivity measured at the study sites using IgG ELISA, was 13.6% overall and with a similar trend at the site level as dengue seroprevalence (data not shown). Its confounding influence is therefore likely to be minimal. Because IgG ELISA is unable to distinguish primary from secondary infections we measured only the rate of primary seroconversion, and are unable to quantify the burden of secondary and subsequent dengue infections.

## Conclusions

We demonstrate high dengue FOI in multiple Indian settings. Observed variations are likely reflective of dengue epidemiological variation in different parts of India. These data may be used for benchmarking the dengue endemicity in other areas in India, and to allow comparisons based on other epidemiological indicators.

## Additional files

Additional file 1: **Table S1.** Number of subjects with IgG data available from DNG10 study according to age (% seropositive). (DOCX 20 kb)

Additional file 2: **Table S2.** Estimates of the number of children experiencing primary dengue infections in 2011, in India overall. (DOCX 18 kb)

## Data Availability

Data sufficient to replicate the current study are provided in Additional file 1. Subject-level data are available from the corresponding author on request.

## Abbreviations

ELISA: Enzyme-linked Immunosorbent Assay
FOI: Force of Infection
IgG: Immunoglobulin G
JE: Japanese encephalitis
PRNT: Plaque reduction neutralization test
