# Supplementary material for: Estimated dengue force of infection and burden of primary infections among Indian children
Source: BMC Public Health. 2019 Aug 14;19:1116. doi: 10.1186/s12889-019-7432-7 (PMC6694619; doi:10.1186/s12889-019-7432-7)
Supplement: Supplementary file 1 — Table S1. Number of subjects with IgG data available from DNG10 study according to age (% seropositive). (DOCX 20 kb) [file 12889_2019_7432_MOESM1_ESM.docx]

**Additional file**

# **Manuscript title: Estimated dengue force of infection, seroprevalence and burden of primary infections among Indian children**

Additional file 1: Table S1. Number of subjects with IgG data available from DNG10 study according to age (% seropositive)

| Age in years | Delhi | Kalyani | Wardha | Mumbai | Hyderabad | Bangalore | Total |
| --- | --- | --- | --- | --- | --- | --- | --- |
| 5 | 106 (43.4) | 60 (15.0) | 54 (46.3) | 31 (51.6) | 107 (33.6) | 67 (61.2) | 425 (40.7) |
| 6 | 49 (57.4) | 62 (14.5) | 51 (50.1) | 57 (68.4) | 111 (50.5) | 66 (60.6) | 448 (50.9) |
| 7 | 116 (64.7) | 68 (26.5) | 54 (63.0) | 62 (79.0) | 120 (58.3) | 68 (58.8) | 488 (58.6) |
| 8 | 125 (67.2) | 76 (26.3) | 55 (81.8) | 61 (90.2) | 122 (73.8) | 55 (70.9) | 494 (67.4) |
| 9 | 107 (73.8) | 43 (27.9) | 53 (81.1) | 60 (88.3) | 109 (73.4) | 43 (62.8) | 415 (70.8) |
| 10 | 94 (73.4) | 14 (50.0) | 56 (89.3) | 30 (96.7) | 70 (58.6) | 22 (63.6) | 286 (73.4) |
| Total | 649 (63.3) | 323 (23.2) | 323 (69.0) | 301 (80.1) | 639 (58.4) | 321 (62.6) | 2556 (59.6) |
