# Supplementary material for: Estimated dengue force of infection and burden of primary infections among Indian children
Source: BMC Public Health. 2019 Aug 14;19:1116. doi: 10.1186/s12889-019-7432-7 (PMC6694619; doi:10.1186/s12889-019-7432-7)
Supplement: Supplementary file 2 — Table S2. Estimates of the number of children experiencing primary dengue infections in 2011, in India overall. (DOCX 18 kb) [file 12889_2019_7432_MOESM2_ESM.docx]

**Additional file**

# **Manuscript title: Estimated dengue force of infection, seroprevalence and burden of primary infections among Indian children**

Additional file 2: Table S2. Estimates of the number of children experiencing primary dengue infections in 2011, in India overall

| Age | Population, 2011 | Proportion experienced primary dengue infection | Number of primary dengue infections |
| --- | --- | --- | --- |
| 0 | 20,311,234 | 0.112 | 2,278,762 |
| 1 | 21,755,197 | 0.212 | 2,166,929 |
| 2 | 23,056,268 | 0.300 | 2,038,870 |
| 3 | 23,974,041 | 0.379 | 1,882,178 |
| 4 | 23,710,038 | 0.448 | 1,652,611 |
| 5 | 26,054,230 | 0.510 | 1,612,262 |
| 6 | 25,654,245 | 0.565 | 1,409,404 |
| 7 | 24,826,640 | 0.614 | 1,210,914 |
| 8 | 26,968,373 | 0.657 | 1,167,802 |
| 9 | 23,424,638 | 0.696 | 900,547 |
| 10 | 20,311,234 | 0.730 | 693,248 |
